# Peptide–Mineral Complexes: Understanding Their Chemical Interactions, Bioavailability, and Potential Application in Mitigating Micronutrient Deficiency

**DOI:** 10.3390/foods9101402

**Published:** 2020-10-02

**Authors:** Xiaohong Sun, Roghayeh Amini Sarteshnizi, Ruth T. Boachie, Ogadimma D. Okagu, Raliat O. Abioye, Renata Pfeilsticker Neves, Ikenna Christian Ohanenye, Chibuike C. Udenigwe

**Affiliations:** 1 School of Nutrition Sciences, Faculty of Health Sciences, University of Ottawa, Ottawa, ON K1H 8M5, Canada; xsun5@uottawa.ca (X.S.); ramin063@uottawa.ca (R.A.S.); rboac063@uottawa.ca (R.T.B.); iohaneny@uottawa.ca (I.C.O.); 2 College of Food and Biological Engineering, Qiqihar University, Qiqihar 161006, China; 3 Department of Food Science and Technology, Faculty of Agriculture, Tarbiat Modares University, Tehran 14115-111, Iran; 4 Department of Chemistry and Biomolecular Sciences, Faculty of Science, University of Ottawa, Ottawa, ON K1N 6N5, Canada; ookag095@uottawa.ca (O.D.O.); rabio069@uottawa.ca (R.O.A.); rpfei069@uottawa.ca (R.P.N.)

**Keywords:** peptide–mineral complex, micronutrient deficiency, mineral supplement, structure–activity relationship, chemical interaction, bioavailability

## Abstract

Iron, zinc, and calcium are essential micronutrients that play vital biological roles to maintain human health. Thus, their deficiencies are a public health concern worldwide. Mitigation of these deficiencies involves micronutrient fortification of staple foods, a strategy that can alter the physical and sensory properties of foods. Peptide–mineral complexes have been identified as promising alternatives for mineral-fortified functional foods or mineral supplements. This review outlines some of the methods used in the determination of the mineral chelating activities of food protein-derived peptides and the approaches for the preparation, purification and identification of mineral-binding peptides. The structure–activity relationship of mineral-binding peptides and the potential use of peptide–mineral complexes as functional food ingredients to mitigate micronutrient deficiency are discussed in relation to their chemical interactions, solubility, gastrointestinal digestion, absorption, and bioavailability. Finally, insights on the current challenges and future research directions in this area are provided.

## 1. Introduction

Minerals such as iron, zinc, and calcium are inorganic substances, some of which play vital biological roles and are essential nutrients for maintaining human health [1,2]. For instance, iron is integrated into erythrocyte hemoglobin, muscle myoglobin, liver ferritin, and several enzymes in tissues. It is required for oxygen transport, short-term oxygen storage, electron transfer, energy transduction, and oxidoreductase activities [3]. Iron deficiency is the most common nutritional disorder worldwide; it is estimated to affect two billion people, with the highest prevalence in infants, children, adolescents, women of reproductive age, and pregnant women [4,5,6]. The consequences of iron deficiency are koilonychia, mood changes, muscle weakness, impaired immunity, and anemia [3,7]. Zinc plays indispensable roles in the metabolic activity of several metalloenzymes, gene expression, the immune system, and human growth [8,9,10]. Zinc deficiency is prevalent in developing countries and has been implicated in the growth retardation of up to two billion people, as well as approximately 800,000 child deaths per year [6,11]. Calcium is the most abundant mineral in the body and accounts for 1.5–2.2% of total body weight; it is involved in skeletal strength maintenance, muscle contraction, neurotransmission, and blood coagulation [5,12,13]. Calcium deficiency results in a reduction in bone mass and bone-related illnesses, such as osteoporosis and rickets, especially in children and older adults [5,13].

Food fortification has been identified by the World Health Organization (WHO) and Food and Agriculture Organization (FAO) as one of the top four strategies for combating micronutrient malnutrition at the global level [14]. Foods often fortified with minerals are cereals and cereal-based products. For example, wheat flour is the most commonly used food vehicle for iron and calcium, while ready-to-eat breakfast cereals and wheat noodles are popular food carriers for zinc [14]. The fortification of staple foods with minerals is simple, affordable and cost-effective and, as such, can improve the nutritional quality of food and provide public health benefits, even in economically poorer regions [15]. Nonetheless, mineral fortification can lead to alterations of the physical and sensory properties of foods [9]. For instance, iron fortificants induce lipid oxidation, increase rancidity and lead to undesired color changes in foods [16]. Likewise, the fortification of calcium salts can cause unwanted changes in the color, texture, and stability of some foods by increasing the cross-linking of food matrix components such as proteins and polysaccharides [14]. Furthermore, some components of the food vehicle can impede the absorption and bioavailability of the fortified minerals. For instance, the phytates contained in cereals and cereal-based products can form insoluble complexes with minerals, thus inhibiting the absorption of minerals in the gastrointestinal tract [7,9,17].

Mineral complexation with some organic compounds, such as amino acid chelators, has been used to reduce their interaction with the food matrix [5,18]. For example, ferrous bis-glycinate resulted in four times higher iron absorption than ferrous sulphate, which was possible because bis-glycinate effectively protected iron from the inhibitory effect of phytate in maize [19]. Due to the prohibitive cost of amino acids, food protein-derived peptides commonly released in vivo or in vitro by enzymatic proteolysis are promising ligands for complexation with divalent metals towards improving mineral bioavailability and mitigating micronutrient deficiency [5,20]. The mineral-chelating properties of peptides are attributed to the structural diversity of their backbone, which contains both the terminal carboxyl and amino groups, and the side chains of amino acid residues [5,9]. To date, a variety of metal-chelating peptides has been generated and identified from different food sources, such as milk, egg, soybean and sea cucumber [21,22,23,24]. Regardless, caseinophosphopeptides (CPPs) are the most extensively studied mineral-chelating peptides that have been successfully applied as food ingredients for mitigating mineral deficiency [20].

Recently, the potential of food-derived peptides as transition metal ligands to enhance iron or calcium absorption was extensively discussed [2]. Another excellent review had a special emphasis on the role of peptide–metal complexes in decreasing the pro-oxidant effect of minerals [5]. Compared to the published works, this review highlights the potential of using peptide–mineral complexes as functional food ingredients towards the mitigation of micronutrient deficiency, with focus on their solubility, gastrointestinal digestion, absorption and bioavailability. Also, this study discusses the methods for the determination of the mineral chelating activities of peptides, the approaches for the preparation, purification and identification of mineral-binding peptides from food proteins, and the structure–activity relationship of the peptides.

## 2. Methods for the Determination of the Mineral-Chelating Activity of Peptides

### 2.1. Iron-Chelating Activity

Colorimetric assays are commonly used to determine the iron-chelating capacity of peptides. Carter [25] first described the use of ferrozine reagent (3-(2-pyridyl)-5,6-diphenyl-1,2,4-triazine-4′,4′′-disulfonic acid sodium salt) for the quantification of iron in human serum, where the absorbance of the magenta-colored Fe^2+^–ferrozine complex is read at 562 nm. Presently, this method has been adapted and widely used for determining the Fe^2+^-chelating capacity of peptides. Recently, the Fe^2+^-chelating capacity of peptides derived from *Acetes japonicus* was successfully assayed using this method, resulting in a maximal iron binding capacity of 177.7 μg Fe^2+^/g protein [26]. Because of the stable complex formed between Fe^2+^ and ferrozine, this method has been used to determine the saturation curve and binding affinity parameters of Fe^2+^ complexation with casein peptides [27].

The o-phenanthroline method, another colorimetric assay variant, can also be used and includes spectrophotometric analysis at 510 nm due to the formation of the ferrous o-phenanthroline complex [28]. As with ferrozine, the absorbance is inversely related to the iron chelating activity of peptides within the hydrolysates. Therefore, the absorbance is expected to decrease as the metal chelating activity of the peptide increases due to a reduction in the concentration of free iron present in the assay solution [29]. Inductively coupled plasma (ICP) spectroscopy is also used to evaluate the iron binding capacity of peptides [30]. ICP is more sensitive than the chemical methods in detecting and quantifying minute amounts of free Fe^2+^ in the assay after chelation by strong peptide ligands.

The thiocyanate colorimetric assay can also be used to quantify the Fe^3+^ chelating capacity of peptides, since the mixture of Fe^3+^ and thiocyanate in solution develops an intense red color that can be detected and quantified at 484 nm. The free Fe^3+^ content, characterized by high absorbance values, can be obtained through standard curve analysis [31]. The Fe^3+^-binding capacity of peptides from scad (*Decapterus maruadsi*) was determined using this method [32].

### 2.2. Zinc-Chelating Activity

Colorimetric assays can also be used to determine the zinc-chelating activity of peptides. For example, 4-(2-pyridylazo) resorcinol and free Zn^2+^ form a red-colored complex whose absorbance can be measured at 500 nm. This approach was used to determine the zinc-chelating capacity of whey protein hydrolysates [24] and rye secalin-derived tripeptides and their analogues [33]. Furthermore, the ethylenediaminetetraacetic acid (EDTA) complexing titration assay, involving the addition of xylenol orange and hexamethylenetetramine, was developed to determine zinc content. In this assay, the level of bound zinc is determined through the production of a white solid after precipitation of the peptide–zinc complexes with ethanol [34]. Atomic absorption spectrometry (AAS) is another commonly used method for measuring the amount of free zinc and determining the zinc-binding capacity of peptides. The successful application of AAS depends on the identification of Zn^2+^ content prior to and after treatment of the zinc-chelating peptides or hydrolysates [35].

### 2.3. Calcium-Chelating Activity

A colorimetric assay using ortho-crescolphthalein as a binding reagent for soluble calcium has been adopted for determining the calcium content of biological specimens, such as plasma and serum [36]. The principle of the ortho-crescolphthalein complex reagent method is that the soluble peptide–Ca^2+^ complex binds to the ortho-crescolphthalein complex reagent to give a maximum absorbance at 570 nm. Thus, calcium-chelating activity is usually determined by the quantity of calcium bound to the peptide on a weight-to-weight basis [37].

Non-colorimetric spectrophotometry has also been used to identify and quantify the calcium chelating capacity of peptides. Originally developed as a method to determine the chelating capacity of CPPs, this assay involves the use of varying concentrations of peptides that bind to free Ca^2+^ [38]. Following complexation, the extent of peptide chelation of calcium ions is elucidated by quantification of Ca^2+^ in the supernatant using AAS [38]. This method has since been modified to incorporate the binding activity of several calcium-chelating peptides, such as those derived from Antarctic krill and tilapia skin gelatin enzymatic hydrolysates, including the use of flame AAS instead [39,40].

## 3. Preparation, Purification, and Identification of Mineral-Binding Peptides from Food

Many peptides are silent in their native food protein structures but become activated upon release and isolation from the parent proteins. The most commonly used method for the release of peptides from food proteins is by exogenous enzymatic hydrolysis using the proteases of interest, or by fermentation. The resulting protein hydrolysates are multicomponent peptide mixtures [41]. Once the hydrolysates demonstrate mineral-binding ability, the next step is to identify and isolate the active peptides responsible for the binding activity. In general, the peptide mixture is subjected to ultrafiltration at a given molecular weight cut-off and the peptides of interest are extracted or captured using immobilized metal affinity chromatography (IMAC). IMAC is the most widely used technique for isolating metal-binding peptides due to its high sensitivity, selectivity, binding capacity and recovery rate [18,42]. Thus, proteins and peptides that form specific reversible complexes with the metal ions of interest are separated by retention in the solid phase of the column as they are non-covalently linked to the metal [18]. The complex is then dissociated into the free mineral-binding peptide by changing the physicochemical properties of the eluting solvent, such as the ionic strength and pH [9]. This step reduces the strength of the interaction between the peptide and the mineral. The peptide fractions can be subjected to a second purification process involving sequential chromatography steps, such as gel filtration and ion exchange chromatography. A further purification step may be conducted using reversed phase high-performance liquid chromatography (RP-HPLC). The sequences of the purified peptides are identified by mass spectroscopy alone or as hyphenated techniques such as liquid chromatography-electrospray ionization mass spectrometry (LC-ESI-MS), ultra-high performance liquid chromatography-tandem mass spectrometry (UHPLC-MS/MS), and matrix-assisted laser desorption/ionization time-of-flight mass spectrometry (MALDI-TOF-MS).

For example, His, Lys and Arg-rich iron-binding peptides were isolated from sugar-cane yeast (*Saccharomyces cerevisiae*) using a combination of membrane ultrafiltration, IMAC and RP-HPLC techniques. The hydrolysates prepared by hydrolyzing sugar-cane yeast with Alcalase, Protex or Viscozyme showed comparable iron-binding capacities, ensured the stability of iron during simulated gastrointestinal digestion and, therefore, enhanced its ability to be dialyzed [43]. Using similar techniques, four iron-chelating casein peptides (EDVPSER, HKEMPFPK, NMAINPSK and AVPYPQR) were isolated from casein hydrolysates and identified using LC-MS/MS [44]. Recently, two iron-binding peptides (DSVNFPVHGL and FKVGQENTPILK) were identified by ultrafiltration and nano-UHPLC-MS/MS from *Acetes japonicus* (shrimp) hydrolysates prepared using Flavourzyme [26]. Likewise, a novel iron-binding 12-mer peptide (QKGTYDDYVEGL) was isolated from *Decapterus maruadsi* (Japanese scad) by a combination of Alcalase hydrolysis, ultrafiltration, IMAC, gel filtration and RP-HPLC. The peptide was suggested to form coordinate complexes with iron, mainly through the carboxyl and hydroxyl oxygen bond, and has been shown to promote the uptake of iron in Caco-2 cells [32].

Zinc-binding peptides have been produced using the targeted (IMAC) approach. Two zinc-chelating peptides (NAPLPPPLKH and HNAPNPGLPYAA) were isolated from defatted wheat germ protein after enzymatic hydrolysis with Alcalase, followed by purification using ultrafiltration, IMAC and macroporous adsorption resin, and identification with MALDI TOF/TOF. HNAPNPGLPYAA demonstrated a high zinc-binding capacity of 92% and improved the in vitro bioavailability of zinc in Caco-2 cells compared to cells treated with ZnSO_4_ [45]. Moreover, a novel 16-mer peptide (HLRQEEKEEVTVGSLK), with a potent zinc-binding capacity (6.56 μg zinc/mg peptide), was purified from oyster protein hydrolysates prepared with pepsin using IMAC and RP-HPLC techniques. The peptide–zinc complex enhanced the stability of zinc during in vitro gastrointestinal digestion. It was proposed that the primary binding sites for zinc were the amino-terminal nitrogen and carboxyl-terminal oxygen atoms of the peptides [46].

**Figure 1:**
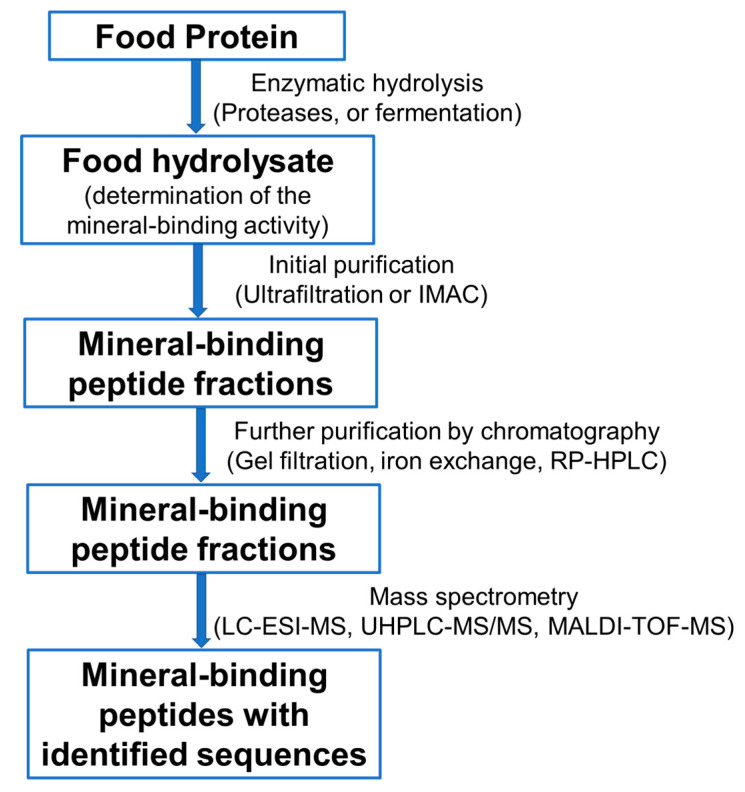
Schematic diagram indicating steps toward the preparation, purification, and identification of mineral-binding peptides from food proteins. IMAC: immobilized metal affinity chromatography; RP-HPLC: reversed-phase high performance liquid chromatography; LC-ESI-MS: liquid chromatography-electrospray ionization mass spectrometry; UHPLC-MS/MS: ultra-high performance liquid chromatography-tandem mass spectrometry; MALDI-TOF-MS: matrix-assisted laser desorption/ionization time-of-flight mass spectrometry.

In a similar way, a high affinity calcium-binding peptide (VLPVPQK) was isolated and identified from casein hydrolysate by RP-HPLC and HPLC-MS/MS. The peptide binds calcium at a stoichiometric ratio of 1:3 and dose-dependently increases the transport and uptake of calcium in Caco-2 cell monolayers [47]. The calcium-binding capacity of the peptides depends on the type of enzyme used, degree of hydrolysis, amino acid composition and molecular weight distribution of the hydrolysates. For instance, peptides isolated from wheat germ protein hydrolyzed with Alcalase at a degree of hydrolysis of 21.5%, or those with a molecular weight less than 2000 Da and those with more hydrophobic amino acids, showed higher calcium-binding affinity [48]. Once the peptide sequences are identified, the structure–activity relationship, the mechanism of binding, and the stability of the peptide–mineral complex can then be investigated. Taken together, the procedures commonly used for the preparation, purification, and identification of mineral-binding peptides from food proteins were illustrated in Figure 1.

## 4. Structure–Activity Relationship and Stability of Peptide–Mineral Complexes

### 4.1. Amino Acid Composition and Peptide Sequence

The amino acid composition and peptide sequence are the most important factors that affect the metal binding capacity of peptides [49]. The major food protein-derived peptide sequences identified to have mineral-binding capacity are summarized in Table 1. Generally, the abundance of structural features such as carboxyl groups, sulfhydryl groups, negative charge, and ionic bonds in peptides improves metal chelation [18]. The His residue of peptides plays an important role in metal chelation [50]. Moreover, the Cys, Glu, Ser and Asp residues, which contain S, O and N atoms, participate in the formation of peptide–mineral complexes [51]. Apart from the amino- and carboxyl-terminal groups, the main amino acid side chain groups that bind with metals are the carboxyl group of Asp and Glu, the imidazole group of His, the hydroxyl group of Ser, the sulphydryl group of Cys, the ε-amino group of Lys and the guanidine group of Arg [49,51,52,53]. For example, the hydroxyl group of Ser has a strong binding potential with zinc [51]. The side chain of cationic residues at physiological pH would cause electrostatic repulsion with the metals; however, Arg forms complexes with several divalent metals depending on its protonation constant [54,55].

Peptides with potent iron-binding activity were purified from β-lactoglobulin and whey protein hydrolysates and found to be rich in Asp, Glu, and Pro [56,57]. In addition, the Asp and His composition of sea cucumber (*Stichopus japonicus*) hydrolysates were thought to be the main chelators of iron. A strong positive correlation was reported between the Asp and His content and iron-binding capacity of peptides; interestingly, no correlation was observed with Cys content [53,63]. Other than the amino acid composition, the peptide sequence is also crucial for metal binding [64]. CPPs are widely used to formulate mineral-enriched infant formula. It was demonstrated that CPPs could increase the bioavailability of zinc, iron, and calcium because of the presence of the highly polar sequences of phospho-Ser and Glu residues [58]. The three continuous phospho-Ser residues played an important role in increasing the calcium binding capacity of the CPP, i.e., Ser(P)-Ser(P)-Ser(P)-Glu-Glu [58]. The presence of an Arg residue at the *C*-terminus has been reported in many peptides with iron-chelating activity [20,65]. Asn residues in peptides may also facilitate their interaction with zinc. A study of rapeseed protein-derived peptides with high zinc binding capacity demonstrated that the presence of an acidic amino acid at the *N*-terminus (e.g., Asn-Ser-Met, Glu-Pro-Ser-His) and basic amino acid at the *C*-terminus (e.g., Ala-Arg, Glu-Pro-Ser-His, Gly-Lys-Arg) cause stronger binding of the peptides to zinc [64]. Furthermore, Wang, Li and Ao [34] compared the zinc-binding capacities of Asn-Cys-Ser and Leu-Ala-Asn and reported that the presence of acidic residues at the *N*-terminus played a more significant role in metal binding than those at the *C*-terminus. Nonetheless, controlled studies with rationally-designed peptides are needed to validate the proposed sequence–function relationships. One available example showed that the sequences of three tripeptides of the same amino acid composition (Cys, Gln, Val) did not significantly influence their zinc-chelating capacity [33].

### 4.2. Molecular Weight, Hydrophobicity, and Peptide Charge

The relationship between peptide molecular weight and their mineral-binding capacity is not well defined. Most purified peptides with a high affinity to iron have a molecular weight of 300–1500 Da [20]. Collectively, dipeptides and tripeptides are the most recommended peptide mineral carriers [26,51,66]. This may be because of their higher aqueous solubility compared to larger peptides, which may aggregate in a metal solution. Torres-Fuentes et al. [63] reported that peptides with a molecular weight <500 Da have stronger iron-chelating activity than larger peptides. Evaluation of hydrolysates from *Acetes japonicus* demonstrated that the 1–3 kDa peptide fraction had the highest iron-binding capacity [26]. However, another study reported that peptides with high molecular weights had better iron binding capacity [67]. This type of peptide promoted mineral uptake in cultured intestinal cells [68] and those with low molecular weights were more stable during in vitro gastrointestinal digestion [69]. In addition, smaller peptides with a higher charge density have a stronger affinity towards metals [66].

The amount of hydrophilic amino acid residues, such as Asp, Glu, Arg and His, is positively correlated with the iron-binding capacity of peptides, whereas peptides with high amounts of hydrophobic amino acids such as Met and Val were reported to be weaker chelators [53]. Hydrophilic peptide fractions of whey protein hydrolysates showed higher iron-binding capacity [70]. Contrastingly, Le Vo et al. [26] purified two iron-binding shrimp peptides with high hydrophobicity scores of 17.8–19.6 and 17.6–21.0 at pH 2 and pH 6.8, respectively. Hydrophobic amino acid residues were reported to be favorable sites for binding to calcium and a positive correlation between the hydrophobic amino acid content of hydrolysates and binding potential was obtained [48]. Because of the lack of detailed experimental evidence, the fundamental basis of such an interaction is currently speculative.

Another important factor that affects the binding capacity is the peptide net charge. Increasing negative charges, such as COO- of sea cucumber (*Stichopus japonicus*) ovum hydrolysates, showed a similar effect of increasing hydrophilic amino acids [53]. Udechukwu et al. [24] reported that whey protein hydrolysates with higher net negative surface charge (zeta potential) resulted in higher zinc-binding capacity. Also, the negative charge was considered an important factor for calcium-binding peptides derived from porcine blood plasma proteins [71]. Notably, the negative surface charge of five casein hydrolysates was strongly correlated (*r*_s_ = 0.90) with their ligand dissociation constant (*K*_d_; represents the strength of the interaction) and not their maximum specific ligand binding (*B*_max_; represents the total metal bound at a given time) [33]. The zeta potential value of the pentapeptide DHTKE changed from positive to negative after binding to Ca^2+^ and it was suggested that the positively-charged moieties may have been involved or shielded during the complexation [59]. Calcium, zinc, and iron chelation was suggested to be affected by the synergistic effect of hydrophobic and hydrophilic charged residues [66]. Taken together, a comprehensive understanding of the relationship between hydrophobicity, the charge of peptides, and their mineral-chelating activity will rely on future studies.

### 4.3. Chemical Interactions Involving in Peptide–Mineral Complex Stability

**Figure 2:**
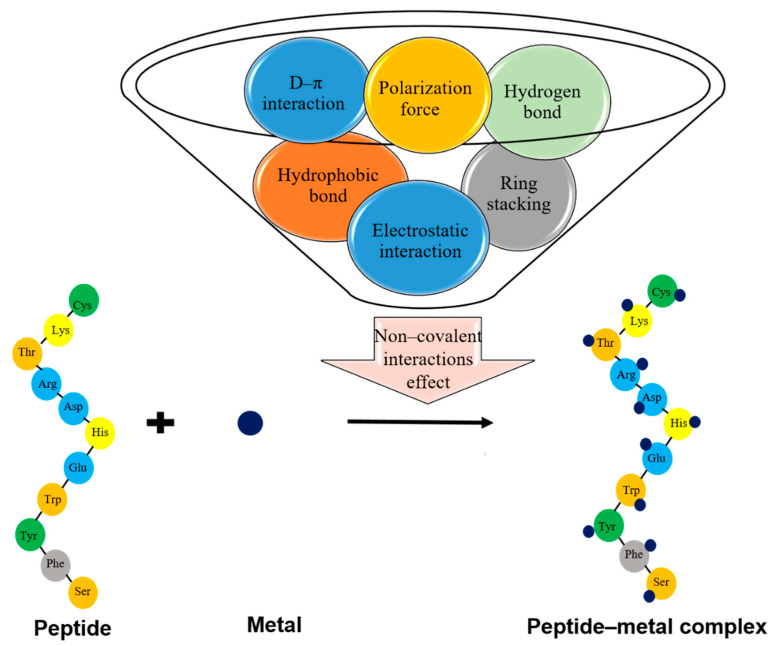
The chemical interactions involved in the formation of peptide–mineral complexes. The amino acids with a high tendency to bind to minerals are illustrated.

The forces involved in the formation and stabilization of peptide–mineral complexes are shown in Figure 2. The side chains of amino acid residues have a significant effect on the stabilization of the conformation of peptide–metal complexes. Primary attraction starts with electrostatic interactions between the metal ions and the negatively charged side chains (Glu, Asp), followed by hydrophobic interactions and ring stacking by aromatic amino acids (Phe, Tyr, and Trp), which stabilize the complex structure [66,72]. In addition, methyl and methylene groups of the coordinating ligand structure improve the stability of the complex. The polarizability of the methyl group and methylene bridge in the peptide contributes to electrostatic and polarization forces and enhances the affinity to iron. The methyl group does not form a hydrogen bond with charged groups, but it is polarizable; the influence of the methyl group on hydrogen bond formation was demonstrated in Thr and Ser [60,73]. Furthermore, changes in the ^1^H NMR chemical shifts and coupling constants of the Cys methine proton in Gln-Cys-Ala in the presence of zinc suggest that it may be actively involved in the formation or stabilization of the peptide–metal complex [33].

Ligand–ligand interactions between amino acid residues surrounding the metal ion and the interaction of aromatic ligands and the metal ion by d–π interaction have significant stabilizing effects on the peptide–metal complex. The d–π interaction is the result of near contact between the aromatic rings of Phe, Tyr, Trp and metal ions [74]. Moreover, polar and charged side chains, such as the guanidinium group of Arg, participate in hydrogen bond formation. Guanidine does not participate directly in metal binding because it is protonated at a wide pH range, but the guanidium group fixes substrates with a negative charge by participation in hydrogen bond formation [72,74]. Some side chain groups, such as the carboxyl group in Asp and Glu residues, the hydroxyl group of Tyr, Thr, and Ser, the thioether of Met and the additional nitrogen atoms of Gln, Asn, Lys and Arg, contribute to increasing the thermodynamic stability of peptide–metal complexes. Among these groups, Asp and Glu residues with a carboxylate-O donor atom have the most significant influence on metal binding [75]. Udechukwu et al. [24] suggested that the side chains of Asp/Glu and Ser/Thr may have participated in the formation of whey protein hydrolysate–zinc complexes. In contrast, the bulky side chain of amino acid residues such as Leu, Trp or Phe were reported to have a negative effect on the thermodynamic stability of the complex [75]. The presence and amount of phospho-Ser/Thr are also crucial in determining the surface negative charge and K_d_ of casein-derived peptides that bind iron by electrostatic interaction [27].

## 5. Solubility, Bioavailability and Absorption of Peptide–Mineral Complexes

In evaluating the efficiency of peptide–mineral complexes as a means of preventing mineral deficiency, it is important to consider the solubility, absorption, and bioavailability of the complexes after oral consumption and gastrointestinal digestion [5]. Metal-coordinated peptide complexes must be soluble in the vehicle to become effective delivery agents and to increase the mineral concentration in the lumen [9,35,76]. Minerals are rendered less reactive and more soluble when they form complexes with peptides. If the peptides withstand hydrolysis by gut proteases, the complexes proceed to the small intestine unmodified for absorption. pH conditions play an important role in the solubility of peptide–mineral complexes. For instance, iron salts exhibited a low solubility of 5.9% at pH 6 and pH 8, whereas peptide–iron complexes produced over 90% iron solubility under the same conditions [77]. In the study, the solubility of the whey protein isolate–iron complexes was higher at gastric and intestinal pH conditions than at pH 4. This is possibly due to the isoelectric point precipitation occurring around pH 4.8 [77]. The low solubility of free iron in the intestine is a significant factor that contributes to its poor bioavailability [78]. Although solubility of the peptide–mineral complex is crucial to ensure bioaccessibility, it does not guarantee the absorption of the minerals. Caetano-Silva et al. [77] found that metal complexes with low molecular weight (<5 kDa) peptides exhibited significantly higher in vitro bioavailability, albeit at similar levels of bioaccessibility as measured by solubility.

The bound metal released from the peptides in the small intestine is desirable for absorption [9]. This means that the binding forces between the mineral and peptide should be able to withhold the mineral amidst the other competitive species present, especially in the stomach, and release it at the absorption site in the intestine [31]. For example, phosphoserine residues found in CPPs can bind weakly to zinc and form complexes which can be gradually released in the small intestine for absorption [76,79]. Highly anionic surfaces of whey peptides resulted in strong chelation and more stable peptide–zinc complexes in the gastric condition, but over 50% of the zinc remained in the complex after intestinal digestion in vitro [9]. Therefore, care must be taken in formulating the peptide–metal complexes to achieve moderate affinity that ensures high gastric stability and effective intestinal release of the minerals.

The molecular weight of peptides plays an essential role in mineral release. Complexes formed with whey peptides of high molecular weights (e.g., >5 kDa) hindered the release and cellular uptake of iron [77]. Likewise, the egg proteins ovotransferrin and phosphovitin formed strong and stable complexes with Fe^3+^, thus impeding iron release and compromising its solubility and ability to dialyze [80]. In addition, side chain groups in peptides could affect the solubility of the released minerals. For example, the sulfhydryl group of Cys can reduce Fe^3+^ to Fe^2+^ to favor the release of iron in its soluble Fe^2+^ form, whereas Fe^3+^ is insoluble at intestinal pH [80,81]. It is important to limit mineral release from the complexes at the acidic stomach pH because the free minerals, i.e., calcium, would subsequently reach the small intestine and form insoluble compounds (e.g., calcium hydroxide) at neutral pH. This would hinder mineral absorption and the insoluble compounds can also accumulate on the intestinal walls and obstruct the absorption of other nutrients [82].

The usual routes of peptide absorption include passive transcellular diffusion, vesicle-mediated intracellular transport of oligopeptides (transcytosis), peptide transporter (PepT1)-mediated permeation, and paracellular transport across the intestinal epithelium [83,84]. Once the peptide–mineral complex reaches the small intestine, it can either be absorbed intact via the usual peptide absorption mechanisms, or the mineral can be dissociated from the complex and absorbed alone. Preformed peptide–iron complexes have been suggested to be absorbed into Caco-2 cells via a similar transport pathway as peptides [77]. This route was corroborated in a study of the cellular uptake of CPP–zinc complexes [85]. The exact mechanism by which the peptide–mineral complexes cross the brush border of enterocytes is unknown. The metals are thought to be released inside the cells with the help of a protein that binds the metals. A similar route is known for heme iron, where an iron-binding protein transports it into the cell, with tight regulation of its release when needed [86].

Favorably, the peptide–mineral complexes mostly remain inert and stable in the gastrointestinal tract until their release later during digestion. The efficiency of the complex to preserve the mineral until release at the absorption site depends on whether complexation occurred prior to or during digestion [5]. The neutral pH of the small intestine encourages the precipitation of the metals, making them less soluble for absorption. The presence of other reactive species and antinutritional factors in the food matrix also reduces the chances of peptide–mineral complex formation. Moreover, protonation of the binding sites of metals due to the gastric pH condition makes spontaneous interactions between metals and peptides less probable [87]. Because of these conditions, food fortification with preformed peptide–mineral complexes is more effective in mitigating mineral deficiencies. Prior complexation gives room to control integral factors and enhance successful delivery of the minerals at the absorption sites.

In vitro cell models are commonly used to evaluate the bioavailability of minerals from peptide–mineral complexes, followed by animal models, but not as much human studies due to the complexity of human trials and potential safety concerns. Due to their similarity with intestinal cells, Caco-2 cells have been more frequently used for in vitro bioavailability studies. As shown in Table 2, the markers of calcium, iron, and zinc bioavailability were improved when preformed peptide–mineral complexes were used. For instance, calcium absorption was significantly increased in Caco-2 when complexed with NDEELNK and sea cucumber ovum hydrolysate [21]. Caetano-Silva et al. [77] also observed ferritin synthesis, resulting in a 70% increase in iron uptake when Caco-2 cells were treated with iron complexes with low-molecular weight (<5 kDa) peptides. The growth media used for in vitro cell transport studies must be devoid of the evaluated minerals. Although this might be necessary to avoid interference with the mineral assay, the depleted levels may promote a higher absorption rate of the mineral, which is not representative of normal absorption. For example, the absorption of iron depends on the iron status of the body, where lower levels stimulate higher absorption rate and vice versa [68]. It is noteworthy that most of the studies were conducted in isolation, with no interference from other components of the food matrix. Furthermore, specific proteases were used to hydrolyze the complexes in most studies instead of subjecting them to a simulated in vitro digestion system that more closely mimics the physiological environment. Hence, it is important to comprehensively assess the interaction of the peptide–mineral complexes with other components of the food matrix along the gastrointestinal tract in order to achieve a pragmatic application of the complexes in mitigating mineral deficiency.

## 6. Sustainable Production of Mineral-Binding Peptides

The feasibility of applying the mineral binding peptides depends on a number of factors. First, it is imperative to consider the sustainability of the system used for the production of peptide–mineral complexes [41]. Most of the studies used traditional food sources, especially animal-derived proteins, and involved extensive processing to generate the mineral-binding peptides. For sustainability, some research has been conducted to prepare metal-chelating peptides from alternative sources, mainly from seafood and crop byproducts [18]. For example, sunflower meal is the major byproduct of oil extraction and copper-chelating peptides have been identified from defatted sunflower meal hydrolysates produced with pepsin and pancreatin [91]. Besides agri-food byproducts, a recent study identified a novel calcium-binding peptide (Glu-Pro-Ala-His) from *Auxis thazard* protein hydrolysates; this protein material is a type of tuna with low nutritional value [62]. Nonetheless, further research efforts are still needed to enhance the sustainable production of metal-chelating peptides using alternative protein sources. Furthermore, the purification of mineral-binding peptides is costly, thus making crude protein hydrolysates the more promising candidates for practical applications in developing food products enriched with peptide–mineral complexes.

## 7. Concluding Remarks and Future Perspectives

Fortification of staple foods such as cereals and cereal-based products with minerals is simple, affordable and cost-effective. Thus, fortification has become a key strategy for combating micronutrient deficiency and improving public health. However, mineral fortification can negatively affect the physical and sensory properties of foods. Peptide–mineral complexes are a promising strategy towards the enhancement of mineral absorption and the reduction of micronutrient deficiency through the development of the complexes as mineral supplements or functional foods.

Colorimetric assays are the most widely used methods for determining the mineral chelating capacity of peptides. However, the presence of co-existing metals or metal-chelating contaminants in the food product or assay matrix would reduce the accuracy of the results, especially by giving false positive results. A variety of metal chelating peptides have been generated from different food sources, such as milk, egg, soybean, rye, and sea cucumber. It would be beneficial if future research could focus on identifying more metal chelating peptides from alternative protein sources, such as edible insects, microalgae and agri-food processing byproducts, which would contribute to achieving a diversified and sustainable agri-food system. The yield of purified mineral-binding peptides is often low and, therefore, new technology is needed for the large scale isolation and purification of the peptides. Moreover, the preparation of crude food protein hydrolysates with mineral-binding capacity is highly recommended in future research to realize the commercialization potential of the complexes. Understanding the structure–activity relationship would shed more light on the chelating mechanisms of minerals and peptides. Despite the availability of studies on the importance of the amino acid composition, there is no consensus on the role of the peptide sequence, molecular weight, hydrophobicity, and charge in mineral binding. This gap further confounds the understanding of the structure–activity relationship. Moving forward, future research should provide more insights into the chemical nature of peptide–mineral complexes, including the specific ligands, their binding sites, binding affinity, and major driving forces. These studies could facilitate the commercialization of peptide–mineral complexes as either mineral supplements or as mineral-fortified functional foods. In addition, human trials are needed in susceptible populations to validate the efficacy of the food-derived peptides in enhancing the mineral bioavailability and mitigating mineral deficiency.

## Figures and Tables

**Table 1:** Metal-binding peptide sequences from different protein sources.

Peptide Sequences	Metal Ions	Source	Location	Net Charge (pH 7.0) *	Molecular Weight *	References
Asp-Ala-Asp-Ser-Val-Asn-Phe-Pro-Val-His-Gly-Leu	Iron	*Acetes japonicus*	n/a	−1.9	1270	[26]
Phe-Lys-Val-Gly-Gln-Glu-Asn-Thr-Pro-Ile-Leu-Lys	Iron	*Acetes japonicus*	n/a	1	1374	[26]
Cys-Gln-Val	Zinc	Rye secalin	324–326	−0.1	348	[33]
Gln-Cys-Ala	Zinc	Rye secalin	343–345	−0.1	320	[33]
Leu-Ala-Gly-Asn-Pro-(Asp)_2_-Glu-Phe-Arg-Pro-Gln	Iron	Defatted walnut flake	n/a	−2	1358	[49]
Val-Gln-Asp-Glu-Leu-Val-Ala-(Val)_2_	Iron	Defatted walnut flake	n/a	−2	971	[49]
Ser-Met	Iron, Zinc	Sesame	n/a	0	236	[51]
Asn-Cys-Ser	Iron, Zinc	Sesame	n/a	−0.1	322	[51]
Tyr-Val-(Glu)_2_-Leu-Lys-Pro-Thr-Pro-Glu-Gly-Asp-Leu-Glu-Ile-Leu	Iron	Bovine β-lactoglobulin	42–57	−4	1845	[56]
Arg-Thr-Pro-Glu-Val-(Asp)_2_-Glu-Ala-Leu-Glu-Lys	Iron	Bovine β-lactoglobulin	124–135	−3	1401	[56]
Phe-Lys-Asp-Leu-Gly-(Glu)_2_-His	Iron	Bovine serum albumin	11–18	−1.9	974	[56]
Lys-(Asp)_2_-Ser-Pro-Asp-Leu-Pro-Lys	Iron	Bovine serum albumin	106–114	−1	1014	[56]
(Asp)_3_-Leu-Thr-(Asp)_2_-Ile	Iron	Bovine α-lactalbumin	82–89	−5	921	[56,57]
Thr-Pro-Glu-Val-(Asp)_2_-Glu	Iron	Bovine β-lactoglobulin	125–131	−4	889	[57]
Ser(P)_3_-(Glu)_2_	Iron, Zinc, Calcium	Bovine β-casein	18–21	−2	777	[58]
Asp-His-Thr-Lys-Glu	Calcium	Chicken egg white	n/a	−0.9	629	[59]
Ser-Val-Asn-Val-Pro-Leu-Tyr	Iron	Barley B1-hordein	275–281	0	791	[60]
Lys-Gly-Asp-Pro-Gly-Leu-Ser-Pro-Gly-Lys	Calcium	Pacific cod bone	n/a	1	955	[61]
Glu-Pro-Ala-His	Calcium	*Auxis thazard*	n/a	−0.9	452	[62]

* Net charges and molecular weights of peptides were calculated using a peptide property calculator (https://pepcalc.com/). Calculated net charges at pH 7.0 differ from experimental charges due to differences in assay pH conditions. n/a—specific protein source and fragment location not available.

**Table 2:** Effect of preformed peptide–mineral complexes on the cellular uptake and bioavailability of minerals.

Type of Study	Peptide/Hydrolysate	Mineral	Treatment Prior Bioavailability Assay	Bioavailability Assay/Markers of Bioavailability	Effect on Bioavailability/Absorption	Reference
*In vitro* using Caco-2 cells	NDEELNK (from trypsin hydrolysis of sea cucumber ovum)	Calcium	*In vitro* digestion	Calcium absorption	Increased calcium absorption	[21]
*In vitro* using Caco-2 cells and HT-29	Sea cucumber ovum hydrolysate (trypsin, Alcalase, Neutrase, papain, Flavourzyme)	Calcium	*In vitro* digestion	Calcium solubility intracellular calcium concentration	Higher calcium solubility intracellular calcium concentration in complexes	
*In vitro* using Caco-2 cells	α-Lactalbumin hydrolysate β-Lactoglobulin hydrolysate (Alcalase, β-LGH )	Iron	*In vitro* digestion	Ferritin content Iron absorption	β-Lactoglobulin hydrolysate–iron complexes significantly improved iron absorption and ferritin	[37]
*In vitro* using Caco-2 cells	SVNVPLY	Iron	*In vitro* digestion	Ferritin formation	Cell uptake increased 4 times after pepsin–pancreatin digestion	[60]
*In vitro* using Caco-2 cells	Whey protein isolate fractionates (pancreatin hydrolysis)	Iron	*In vitro* digestion	Ferritin synthesis in cell culture model	Ferritin synthesis in complexes with low-molecular weight (<5 kDa)	[77]
*In vitro* using Caco-2 cells	Caseinophosphopeptides (CPPs) (β-CN(1–25)4P, αs1-CN(64–74)4P and αs2-CN(1–19)4P)	IronZinc	*In vitro* digestion	Ferritin synthesis	Increased ferritin synthesis Increased zinc uptake	[85]
*In vitro* using Caco-2 cells	GPAGPHGPPG from Alaska pollock skin	Calcium Iron Zinc	Hydrolysis with pepsin	Transport in Caco-2 cell monolayer	112.7% increase in calcium transport 27.7% increase iron 32.3% in zinc transport	[88]
*In vivo* (iron-deficiency anemia male rats)	Duck egg white peptides (neutrase)	Iron	Feeding	Hematological test, serum iron, serum ferritin	Hematology levels increased to the normal levels by peptide–iron complexes	[22]
*In vivo* (iron-deficiency anemia female rats)	β-Lactoglobulin hydrolysate (Alcalase)	Iron	Feeding	Hematological test Serum ferritin and transferrin	β-Lactoglobulin hydrolysate–iron complex significantly improved serum iron level, total iron-binding capacity and transferrin saturation, serum ferritin	[68]
*In situ* single-pass intestinal perfusion (in Wistar rats)	Pacific Cod (*Gadus macrocephalus*) Bone calcium binding peptides (trypsin and neutral protease)	Calcium	Single-pass intestinal perfusion	Calcium absorption Calcium retention	Increased calcium absorption and serum calcium	[89]
*In vivo* (iron-deficiency anemia male rats)	Tripeptide REE	Iron	Feeding	Hematological test Serum ferritin and transferrin Serum iron Hepcidin mRNA expression	Increase in hematological parameters to normal levels Restoration of renal coefficient, total iron-binding capacity, and transferrin, liver hepcidin mRNA to normal levels	[90]
